# Insights into the Mode of Action of the Sactibiotic Thuricin CD

**DOI:** 10.3389/fmicb.2017.00696

**Published:** 2017-04-20

**Authors:** Harsh Mathur, Vincenzo Fallico, Paula M. O’Connor, Mary C. Rea, Paul D. Cotter, Colin Hill, R. Paul Ross

**Affiliations:** ^1^Moorepark Food Research CentreCounty Cork, Ireland; ^2^APC Microbiome Institute, University College CorkCork, Ireland; ^3^School of Microbiology, University College CorkCork, Ireland

**Keywords:** bacteriocin, mode of action, cytometry, membrane potential, viability

## Abstract

Thuricin CD is a two-component bacteriocin, consisting of the peptides Trnα and Trnβ, and belongs to the newly designated sactibiotic subclass of bacteriocins. While it is clear from studies conducted thus far that it is a narrow-spectrum bacteriocin, requiring the synergistic activity of the two peptides, the precise mechanism of action of thuricin CD has not been elucidated. This study used a combination of flow cytometry and traditional culture-dependent assays to ascertain the effects of the thuricin CD peptides on the morphology, physiology and viability of sensitive *Bacillus firmus* DPC6349 cells. We show that both Trnα and Trnβ are membrane-acting and cause a collapse of the membrane potential, which could not be reversed even under membrane-repolarizing conditions. Furthermore, the depolarizing action of thuricin CD is accompanied by reductions in cell size and granularity, producing a pattern of physiological alterations in DPC6349 cells similar to those triggered by the pore-forming single-component bacteriocin Nisin A, and two-component lacticin 3147. Taken together, these results lead us to postulate that the lytic activity of thuricin CD involves the insertion of thuricin CD peptides into the membrane of target cells leading to permeabilization due to pore formation and consequent flux of ions across the membrane, resulting in membrane depolarization and eventual cell death.

## Introduction

Bacteriocins are ribosomally synthesized peptides that exert antimicrobial effects predominantly against closely related bacteria, often in the nanomolar range (Cotter et al., 2013). Bacteriocins are broadly classified into class I (post-translationally modified) and class II (unmodified) bacteriocins. The mode of action of the most commonly studied subclass of bacteriocins, the lantibiotics, involves the peptide(s) binding to lipid II, followed by pore formation in some cases. Examples of such lantibiotics include Nisin A and lacticin 3147 (Wiedemann et al., 2006; Field et al., 2010). In contrast, the mode of action of several class II bacteriocins involves the mannose-phosphotransferase system as a receptor (Oppegård et al., 2007). The mechanism of action of Gram negative bacteriocins such as microcins often involves targeting DNA gyrase, RNA polymerase or Asp-tRNA synthetase (Cotter et al., 2013). Recently, the mode of action of the class II bacteriocin, lactococcin G, has been identified and shown to involve the UppP protein as a receptor (Kjos et al., 2014). In another recent study, sequencing of mutants displaying attenuated sensitivity to plantaricin JK revealed a putative amino acid transporter as a possible receptor for this class II bacteriocin (Oppegård et al., 2016).

The sactibiotics are a relatively newly designated subgroup of bacteriocins and include subtilosin A, thurincin H, propionicin F, and thuricin CD (Arnison et al., 2013). With the exception of subtilosin A, the sactibiotics described thus far are narrow spectrum antimicrobials. Significantly, the peptides are relatively hydrophobic in nature and their 3D structures tend to be hairpin-shaped. The hydrophobic residues generally point outward, indicating that these residues most likely interact with the hydrophobic membrane of target strains. With respect to elucidating the mode of action, there have been relatively few studies conducted with the sactibiotic subclass of bacteriocins thus far. The most comprehensive study of this nature involved the sactibiotic subtilosin A by Thennarasu et al. (2005) in which the authors described the interaction of the hydrophobic residues of the peptide with the hydrophobic lipid bilayers of the target membrane. Thuricin CD is one such example of a narrow-spectrum sactibiotic bacteriocin and is composed of two peptides, the 2763Da Trnα and 2861Da Trnβ, predominantly targeting spore-forming Gram positive bacteria including all *Clostridium difficile* strains tested (Rea et al., 2010). Both Trnα and Trnβ also exhibit a characteristic sactibiotic hairpin-like structure generated by three sulfur-to-α-carbon linkages and are relatively hydrophobic, similar to other sactibiotics described (Sit et al., 2011; Arnison et al., 2013). An unusual feature of thuricin CD is that Trnα and Trnβ are similar in sequence and structure, with 45.3% sequence similarity and 39.6% identity. This relatively high percentage sequence and structural similarity suggests that they may share a common receptor/binding site and may have a similar function. However, it is clear that their actions are synergistic (Rea et al., 2010). This high level of similarity is in contrast to other two-component bacteriocins such as lacticin 3147, in which case the associated peptides, Ltnα and Ltnβ, share no homology and are completely different in structure to each other. These differences reflect different functions in that Ltnα binds to lipid II and Ltnβ causes pore formation (Wiedemann et al., 2006). Previous investigations had indicated that thuricin CD may be a slow-acting bacteriocin relative to lacticin 3147. This was determined by quantifying the release of the intracellular enzyme acetate kinase from thuricin CD-sensitive *C. difficile* cells subsequent to exposure to the bacteriocin, and the results demonstrated that the rate of release of this enzyme was relatively slow compared to lacticin 3147-treated cells. This comparatively slow action, along with its narrow-spectrum nature, indicated that the mode of action of thuricin CD is likely to differ from that of the broad-spectrum lantibiotic, lacticin 3147 (Rea et al., 2010).

The traditional culture-dependent analysis of cell viability following antimicrobial challenge can be complemented by flow cytometry (FC) in order to evaluate alterations in cell morphology and physiology and to gain deeper insights into the antimicrobial action of bacteriocins. Supported by an ever increasing catalog of fluorescent probes enabling multi-parametric assessment of bacterial physiology, FC allows us to assess the impact of antimicrobials on the bacterial host in real-time at both population and single-cell level. Studies using FC to investigate the impact of bacteriocins on the cells have generally utilized a combination of probes enabling the assessment of changes in membrane integrity (e.g., Syto 9 and PI), membrane potential [e.g., DiBAC_4_(3), DiOC_2_(3)] and intracellular metabolic activity (e.g., cFDA) (Ratinaud and Revidon, 1996; Ueckert et al., 1997, 1998; Swarts et al., 1998; Martínez et al., 2000; Shapiro, 2000; Budde and Rasch, 2001; Dalmau et al., 2002; Weeks et al., 2006; Gou et al., 2010; Ayari et al., 2012; Pal and Srivastava, 2014; Chopra et al., 2015). The ability to study individual cellular events and to distinguish them from the overall population response makes FC one of the most appropriate tools to provide real-time information regarding the potentially heterogeneous response of a bacterial population to a bacteriocin.

In this study, we attempted to elucidate the mode of action of thuricin CD by using FC to detect structural and physiological changes in sensitive *Bacillus firmus* DPC6349 cells upon exposure to individual, combined or sequentially added Trnα and Trnβ. We hypothesized that the cell membrane is a likely target for thuricin CD peptides, and thus we used real-time FC to monitor the time course modifications in size, complexity and membrane potential of sensitive cells. In addition, we compared the time point viability data obtained by FC analysis of membrane integrity with those generated by traditional growth-based assays. Notably, we found that the thuricin CD peptides elicit depolarization of the membrane, most likely as a consequence of the pore-forming ability of the peptides triggering the movement of ions across the membrane, and this occurs with reductions in cell size and granularity.

## Materials and Methods

### Thuricin CD Purification

Thuricin CD for these assays was purified as described previously, with minor alterations (Rea et al., 2010). Briefly, BHI broth was initially clarified by passing through XAD-16 beads (Sigma Aldrich) prior to autoclaving. Ten ml cultures of *B. thuringiensis* DPC6431 (thuricin CD-producing strain) were grown in BHI broth at 37°C with vigorous agitation and the strain subcultured two times in BHI broth prior to inoculating 1.5 L of clarified BHI broth. The 1.5 L culture was grown overnight for 16 h at 37°C with agitation. The overnight culture was centrifuged at 8260 *g* for 15 min and both the supernatant and cell pellet were retained for further use. The cell pellet was resuspended in 275 ml of 70% IPA, 0.1% TFA and stirred for 4.5 h at 4°C. The supernatant was passed through fresh XAD-16 beads in a column and then washed with 500 ml of 40% ethanol. Thuricin CD was eluted in 400 ml of 70% IPA, 0.1% TFA and this elute was called S1. The cell pellet which was resuspended in 70% IPA, 0.1% TFA was centrifuged at 8260 *g* for 15 min and the supernatant (S2) was added to S1. The IPA was subsequently removed by subjecting the S1–S2 mix to rotary evaporation (Buchi) and the sample was passed through a Phenomenex C-18 column which had been pre-equilibrated with methanol and water just before use. The column was washed with 120 ml of 40% ethanol and thuricin CD eluted in 60 ml of 70% IPA, 0.1% TFA. This was further concentrated by removing the IPA using rotary evaporation, before separating and purifying the two peptides using RP-HPLC. Nisin A and lacticin 3147 were purified as already described (Field et al., 2010; Draper et al., 2013).

### Minimum Inhibitory Concentration (MIC) Assays

In this study, *B. firmus* DPC6349 was utilized as a surrogate thuricin CD-sensitive host, *in lieu* of thuricin CD-sensitive *C. difficile* strains. The aerobic growth conditions of DPC6349, in conjunction with its class I status, facilitated the FC experiments in this study. MIC assays were conducted as previously described with minor modifications (Mathur et al., 2013). Briefly, cells of the bacteriocin-sensitive strain *B. firmus* DPC6349 were grown overnight for 16 h in LB broth at 37°C with vigorous agitation (150 rpm). The following day, the cells were subcultured until they reached mid-logarithmic phase (an OD_600_ of approximately 0.5). At this point, cells were diluted such that the final inoculum for MIC assays was approximately 5 × 10^5^ cfu/ml. Thuricin CD peptides, Nisin A and lacticin 3147 peptides were resuspended in LB broth. The potassium ionophore, valinomycin, was initially reconstituted in DMSO (Molecular Probes) prior to diluting in PBS and subsequently diluting in LB broth. All antimicrobials were finally diluted in LB broth to give the desired starting concentrations and two-fold serial dilutions were performed in 96-well microtitre plates, for MIC assays. MIC readings were taken at 18 h and the MIC was defined as the lowest concentration of the antimicrobial at which there was no visible growth of the target strain. Assays were conducted in triplicate.

### Time Course FC Analyses of Membrane Potential and Morphology in the Bacterial Host during Bacteriocin Challenge

Used alone or in combination with fluorescent probes, FC enables the assessment of various structural and functional cell properties, often leading to a deeper characterization of the physiological heterogeneity of a microbial population (Díaz et al., 2010). Membrane potential in microbial cells can be detected by FC following staining with fluorescent lipophilic cell-permeant probes such as DiOC_2_(3). This carbocyanine dye exhibits red fluorescence in bacterial cells maintaining a large membrane potential and a fluorescence shift toward green emission as a result of membrane depolarization (Novo et al., 1999). Conversely, fluorescence-independent monitoring by FC of the forward (FSC) and side-angle (SSC) light scattering properties of a bacterial population provides useful information about morphological parameters such as cell size and internal granularity/complexity, respectively (Díaz et al., 2010; Shapiro, 2015). The impact of the thuricin CD peptides, Trnα and Trnβ, on the membrane potential and morphology of sensitive *B. firmus* DPC6349 cells was therefore assessed by FC as follows. Cells from a 100 μl aliquot of an overnight culture were recovered by centrifugation (8260 *g* for 10 min), diluted 10-fold in 1 ml of LB broth and activated by incubation at 37°C for 30 min. At this point, the cells were diluted a further 25-fold and stained for 30 min with a solution of 6.3 μM DiOC_2_ (3) (Thermo Fisher Scientific), prepared in PBS and containing 0.21% glucose to energize the cells and thereby generate a large membrane potential. Aliquots of 1.4 ml of stained samples were acquired at medium flow rate (35 μl/sec) using a BD Accuri C6 flow cytometer (Becton Dickinson, Belgium) and a threshold of 10000 on FSC to minimize background. Acquisition and analysis of DiOC_2_(3) fluorescence (FL3, > 670 nm long pass), cell size (FSC) and cell complexity/granularity (SSC) were carried out using the BD Accuri C6 software v. 1.0.2 (Becton Dickinson, Belgium). The microbial population was identified based on its light scattering properties on FSC versus SSC bi-plots and a gating strategy was used to discriminate it from background particles and cell debris. The time course changes in membrane potential and morphology of DPC6349 cells following challenge with an antimicrobial were assessed using the “Run Unlimited” function of the C6 flow cytometer. Unchallenged DiOC_2_(3)-stained cells were initially acquired for 2 min to establish baseline levels of membrane potential and morphological parameters. Subsequently, a small aliquot of a 10–15x stock solution of an antimicrobial in PBS was added and its effect on the physiology of the sensitive target monitored over 15–25 min. The impact on the host’s membrane potential of individual, combined and sequentially added thuricin CD peptides, Trnα or Trnβ, was compared to those exerted by the proton motive force (PMF)-depleting agents Nisin A (Bruno et al., 1992), lacticin 3147 (McAuliffe et al., 1998), and valinomycin (Shapiro, 2004). Stock solutions of valinomycin were prepared in DMSO, whereas diluted working solutions were made using PBS. To generate high external potassium conditions and trigger the membrane depolarizing activity of valinomycin (Shapiro, 2004; Cronin, 2015), 150mM KCl was added to the PBS used to prepare the DiOC_2_(3) staining solution. Assays were conducted in duplicate.

### Light Microscopy to Assess the Impact of Thuricin CD on DPC6349 Cells

Overnight cultures of *B. firmus* DPC6349 cells were diluted 100-fold to yield a starting concentration of approximately 10^7^ cfu/ml for light microscopy analysis. Cells were treated with either 1 μM thuricin CD, 18 μM Trnα, 18 μM Trnβ or PBS without any peptide (negative control) in a final volume of 1 ml. Fifty microliter aliquots were taken at time points 1, 3, and 5 h for microscopy. A light microscope (Zeiss, Axioskop) was utilized to evaluate the effects of the thuricin CD peptides on the morphology and size of DPC6349 cells. Cells were magnified 1000× and images captured with Zeiss AxioCam ERc5s.

### Assessment of Bacterial Survival to Bacteriocin Challenge by FC Analysis of Membrane Integrity and Conventional Plate Count Method

Overnight cultures of DPC6349 were diluted to a final concentration of approximately 10^7^ cfu/ml as described for the membrane potential experiments above, with the exception that LB broth was replaced by PBS as the dilution buffer. To assess the effects of individual and combined thuricin CD peptides, diluted DPC6349 cells were exposed to known equimolar concentrations of Trnα, Trnβ or both peptides and incubated at 37°C with vigorous agitation (400rpm). To analyze the impact of the sequential addition of the thuricin CD peptides, diluted DPC6349 cells were initially exposed to sub-lethal concentrations (1 μM) of either Trnα or Trnβ for 1 h at 37°C to allow the peptide to adsorb to the cell surface. After this time, cells were recovered by centrifugation (8260 *g* for 10 min), washed with PBS to remove any unbound peptide and resuspended in a PBS solution containing a lethal concentration (12 μM) of the second peptide. Negative controls consisted of cells incubated in the absence of any peptide and challenged and unchallenged cells were sampled (100 μl) at various time points (0, 3, 5, and 21 h) and serially diluted in PBS. FC analysis of membrane integrity was carried out by staining appropriate dilutions (i.e., providing less than 1000 cellular events per min to avoid coincidence at the flow cytometer) of each sample with 6.68 μM Syto 9 and 40 μM PI from the Live/Dead^®^ BacLight^TM^ viability kit (Molecular Probes, USA) for 15 min at 37°C, under agitation (400 rpm) in the dark. While Syto 9 enters all cells regardless of their membrane integrity status, PI usually only enters cells with a permeabilized or compromised membrane and, due to its higher binding affinity for nucleic acids, displaces Syto 9. Consequently, live cells with an intact membrane generally exhibit green fluorescence as they only contain Syto 9 whereas dead cells with a compromised membrane are stained red by PI. Injured cells with a permeabilized membrane will contain both dyes in a ratio proportional to the degree of membrane damage and will exhibit orange fluorescence (Shapiro, 2004, 2015). Stained samples were analyzed for 1 min at medium flow rate (35 μl/sec) using a BD Accuri C6 flow cytometer (Becton Dickinson, Belgium) and a threshold of 10000 on FSC to minimize background. Acquisition and analysis of Syto 9 (FL1) and PI fluorescence (FL3, > 670 nm long pass) were carried out using the BD Accuri C6 software v. 1.0.2 (Becton Dickinson, Belgium). The microbial population was identified based on its light scattering properties on FSC versus SSC bi-plots and a gating strategy was used to discriminate it from background particles and cell debris. Assays were conducted in duplicate. For viable plate counts, samples were prepared as described above with serial dilutions conducted using PBS and dilutions plated on LB agar to determine cfu/ml. At least three independent replicates were used and significant differences assessed using Student’s *t*-test for viable plate counts.

## Results

### Assessment of the Minimum Inhibitory Concentrations (MIC) of Various Antimicrobials against *Bacillus firmus* DPC6349

The MIC of thuricin CD against DPC6349 was found to be approximately 24-fold lower than the MICs of the individual peptides Trnα or Trnβ present on their own. In terms of molar values, the MIC of lacticin 3147 was found to be four-fold lower than that of thuricin CD, whereas the MIC of Nisin A was approximately 2.5-fold higher than thuricin CD. Finally, the MIC of the PMF-altering reagent valinomycin was five-fold higher than thuricin CD and the MIC of the antibiotic chloramphenicol was 60-fold higher than thuricin CD, again when expressed in molar values (**Table 1**).

**Table 1:** Minimum inhibitory concentrations of various antimicrobials against *B. firmus* DPC6349.

Antimicrobial	MIC μM/(μg/ml)
Thuricin CD	0.125 **(0.704)**
Trnα	3 **(8.29)**
Trnβ	3 **(8.58)**
Nisin A	0.312 **(1.046)**
Lacticin 3147	0.031 **(0.191)**
Chloramphenicol	7.5 **(2.423)**
Valinomycin	0.625 **(0.69)**

*MIC values expressed in μg/ml are indicated in parentheses in bold.*

### DiOC_2_(3) Enables Detection of Membrane Depolarization and Repolarization in *B. firmus* DPC6349 Cells

**FIGURE 1:**
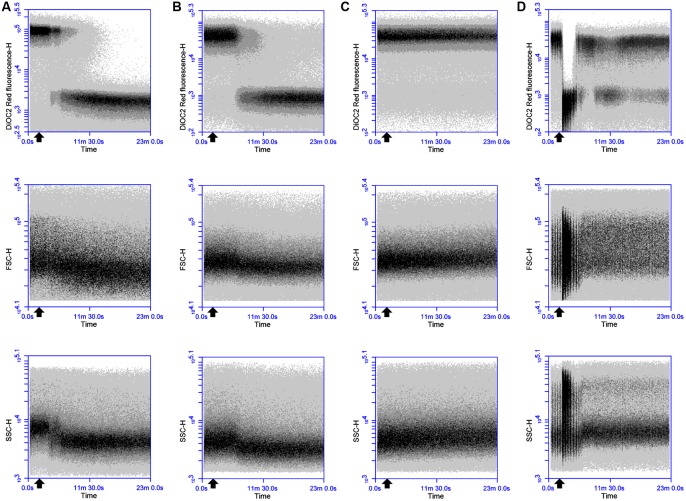
**Effects of Nisin A, lacticin 3147, chloramphenicol and valinomycin on the membrane potential, cell size and cell granularity of *B. firmus* DPC6349: The probe DiOC_2_(3) was used to ascertain the effects of (A)** 3 μM Nisin A, **(B)** 93 nM lacticin 3147, **(C)** 24 μM chloramphenicol, and **(D)** 156 nM valinomycin. A reduction in DiOC_2_(3) fluorescence indicates membrane depolarization whereas decreases in FSC-H and SSC-H values indicate decreases in cell size and cell granularity, respectively. The black arrow depicts the point at which the respective antimicrobials were added.

Dissipation of membrane potential and overall PMF is an essential component of the lytic action of various membrane-active antimicrobial peptides (Bruno et al., 1992; Maftah et al., 1993). As the magnitude of these membrane polarization changes can vary between bacteria (Novo et al., 1999; Shapiro, 2004), the ability of the probe DiOC_2_(3) to detect depolarizing and repolarizing alterations in the membrane potential of DPC6349 cells was assessed using antimicrobials with known mechanisms of action. Preliminary tests revealed a depolarized membrane in 16 h-grown cells, probably as a result of the limiting nutrient conditions typical of stationary phase cells. Consequently, glucose was added to the DiOC_2_(3) staining solutions in order to energize the cells and favor the restoration of a resting basal membrane potential. Nisin A is known to fully dissipate the membrane potential and pH gradient in energized cells, leading to the complete collapse of the PMF (Bruno et al., 1992), and was therefore used to test assay functionality. FC analysis showed that DiOC_2_(3) red fluorescence accumulated to approximately 5 logs arbitrary units in energized DPC6349 cells (**Figure 1A**). Two minutes after the membrane potential baseline was established, cells were exposed to 3 μM (approximately 10x MIC) of Nisin A. A very fast membrane depolarization occurred within 4 min of adding the peptide, as revealed by a major reduction in red fluorescence emission of DiOC_2_(3)-stained cells. Interestingly, the onset of membrane depolarization was accompanied by decreases in cell size (FSC) and granularity/complexity (SSC), probably as a consequence of cell lysis (**Figure 1A**). Similar changes in cell physiology were triggered by the application of 93 nM (approximately 3x MIC) of lacticin 3147 (**Figure 1B**). Lacticin 3147 is a two-peptide lantibiotic bacteriocin which has also previously been shown to alter the membrane potential in sensitive *Lactococcus lactis* subsp. *cremoris* HP cells (McAuliffe et al., 1998). In contrast, no membrane depolarization and reduction in cell size was elicited by the addition of 24 μM of chloramphenicol (approximately 3x MIC) (**Figure 1C**). Chloramphenicol is known to exert its antimicrobial action by inhibiting protein synthesis without causing any alteration in the membrane potential of target cells (Novo et al., 2000; Schifano et al., 2013). Finally, the FC assay was tested using valinomycin, which is a potassium ionophore able to depolarize or hyperpolarize the bacterial membranes under conditions of either high or low environmental potassium, respectively (Shapiro, 2004). In the presence of high concentrations (150 mM) of extracellular KCl, addition of 156 nM (0.25x MIC) valinomycin resulted in an immediate and sharp membrane depolarization, probably caused by a rapid influx of K^+^ ions into the cells (**Figure 1D**). Most of the microbial population reacted to this forced depolarization and was able to repolarize the membrane after a few minutes. This was most likely mediated by the activation of an efflux pump leading to the extrusion of the excess intracellular K^+^ ions which may have accumulated inside the cells. However, a percentage of the population lost the ability to maintain a constant ionic equilibrium at the two sides of the membrane and remained in a depolarized state (**Figure 1D**). As expected at this sublethal concentration (0.25x MIC), no reduction in cell size or granularity accompanied the membrane depolarization triggered by valinomycin (**Figure 1D**).

### Thuricin CD Peptides Permeabilize and Depolarize the Membrane of *B. firmus* DPC6349

**FIGURE 2:**
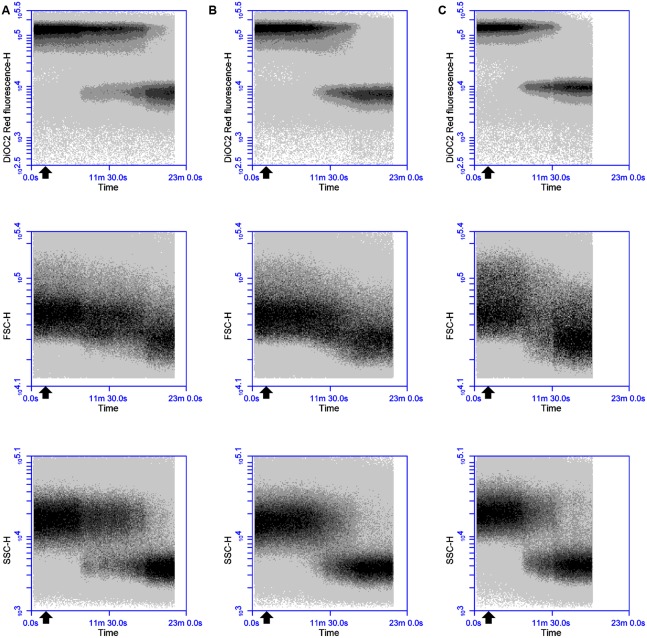
**Effects of 3 μM thuricin CD peptides on the membrane potential, cell size and cell granularity of *B. firmus* DPC6349: The dye DiOC_2_(3) was used to evaluate the effects of (A)** 3 μM Trnα, **(B)** 3 μM Trnβ, and **(C)** 3 μM thuricin CD. The black arrow depicts the point at which the thuricin CD peptides were added.

**FIGURE 3:**
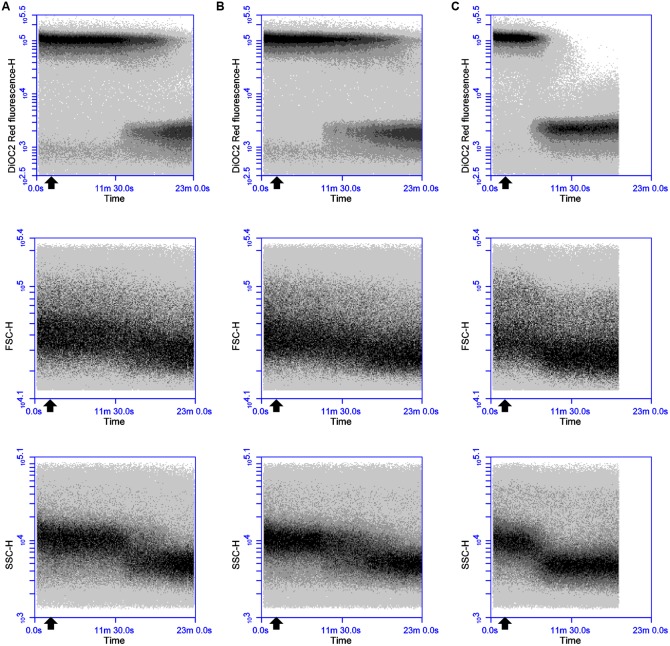
**Effects of 9 μM thuricin CD peptides on the membrane potential, cell size and cell granularity of *B. firmus* DPC6349: The effects of (A)** 9 μM Trnα, **(B)** 9 μM Trnβ, and **(C)** 9 μM thuricin CD were assessed by using the probe DiOC_2_(3). The black arrow depicts the point at which the thuricin CD peptides were added.

In an attempt to elucidate the mode of action of thuricin CD, DiOC_2_(3) staining and real-time FC were used to monitor the time course impact of equimolar concentrations of individual and combined thuricin CD peptides on the membrane potential of *B. firmus* DPC6349 (**Figures 2A–C**). When tested at the MIC of individual peptides (3 μM), both individual peptides triggered membrane depolarization accompanied by reductions in cell size (FSC) and internal granularity (SSC) (**Figures 2A,B**). These patterns of physiological changes in the sensitive host were similar to those exerted by Nisin A (**Figure 1A**) and lacticin 3147 (**Figure 1B**), thereby suggesting that the individual thuricin CD peptides are able to permeabilize and depolarize the membrane of target cells. The combined thuricin CD peptides (**Figure 2C**) were also found to depolarize the membrane and elicited similar decreases in cell size and cell granularity as the individual peptides. In contrast to the lantibiotics Nisin A and lacticin 3147 (**Figures 1A,B**) which triggered very sharp changes in depolarization, the thuricin CD peptides appeared to cause a more gradual depolarization (**Figures 2A–C**). The thuricin CD peptides were also tested at concentrations three-fold their MIC (9 μM) (**Figures 3A–C**) and at these concentrations, it was found that the combined peptides (**Figure 3C**) caused faster depolarization (approximately 5 min after addition of the peptides) compared to the initiation of depolarization approximately 10 min after addition of 9 μM Trnα (**Figure 3A**) and approximately 7 mins after the addition of 9 μM Trnβ (**Figure 3B**). A greater degree of depolarization was apparent at these higher concentrations, compared to lower concentrations of 3 μM (**Figures 2A–C**).

### Membrane Depolarization Is an Integral Part of the Lytic Action of Thuricin CD

**FIGURE 4:**
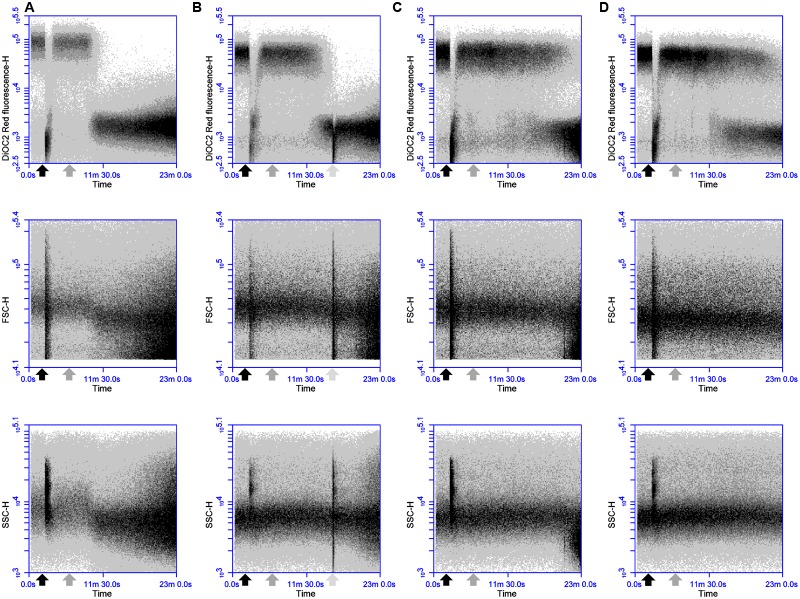
**Effects of the potassium ionophore valinomycin, alone and in combination with thuricin CD peptides: (A)** 156 nM valinomycin (0.25x MIC; black arrow) followed by 18 μM thuricin CD (dark gray arrow) to ascertain its effects on the membrane potential of DPC6349; **(B)** 156 nM valinomycin (0.25x MIC; black arrow) followed by 18 μM thuricin CD (dark gray arrow), followed by a second addition of 156 nM valinomycin (light gray arrow); **(C)** 156 nM valinomycin (0.25x MIC; black arrow) followed by 18 μM Trnα (dark gray arrow); and **(D)** 156 nM valinomycin (0.25x MIC; black arrow) followed by 18 μM Trnβ (dark gray arrow).

Sequential challenge experiments were conducted to determine whether valinomycin can interfere with the ability of thuricin CD peptides to affect membrane potential (**Figure 4**). DPC6349 cells were stained with DiOC_2_(3) in the presence of 150 mM environmental K^+^ ions to favor the depolarizing activity of valinomycin and analyzed by real-time FC. Two minutes after the membrane potential baseline was established, addition of 156 nM valinomycin triggered membrane depolarization-repolarization events (**Figure 4A**) similar to those previously described (**Figure 1D**). Once the cells had restored their resting membrane potential, 18 μM thuricin CD was added and its effects monitored over time (**Figure 4A**). Within approximately 5 min, the sactibiotic caused full membrane depolarization and this effect could not be reversed even after further addition of 156 nM valinomycin (**Figure 4B**). Despite the presence of valinomycin and conditions favoring membrane repolarization due to potential cellular uptake of K^+^, thuricin CD’s impact on the DPC6349 membrane was such that cells were unable to counteract the depolarizing action of the sactibiotic. Again, decreases in cell size (FSC) and granularity (SSC) were observed in parallel with the onset of membrane depolarization, further suggesting this latter event as being an integral part of the lytic activity of thuricin CD (**Figures 4A,B**). Similar experiments conducted using each individual thuricin CD peptide showed that both Trnα and Trnβ were also able to force membrane depolarization in DPC6349 cells despite the repolarizing capacity of valinomycin (**Figures 4C,D**). However, the time course depolarization caused by the individual peptides was slightly slower with 18 μM Trnα causing depolarization approximately 9 min after the addition of the peptide, while 18 μM Trnβ elicited depolarization approximately 7 min after the addition of the peptide. This slower depolarizing effect compared to the combined peptides is in agreement with initial observations (**Figures 3A–C**).

**FIGURE 5:**
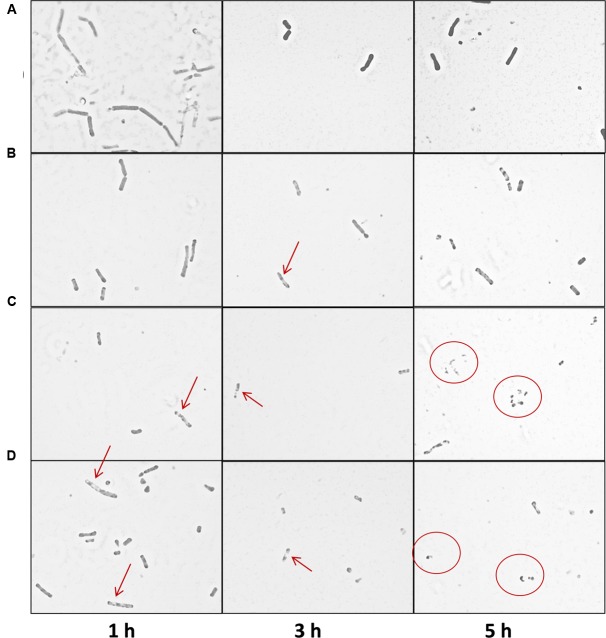
**Compound microscope image analysis of *B. firmus* DPC6349 cells upon exposure to thuricin CD peptides: (A)** DPC6349 cells in PBS; **(B)** DPC6349 cells exposed to 1 μM thuricin CD; **(C)** DPC6349 cells treated with 18 μM Trnα; **(D)** DPC6349 cells treated with 18 μM Trnβ. Time points 1, 3, and 5 h post-exposure are shown. The red arrows depict regions of the membrane which have become less dense due to the peptides. Smaller, fragmented cells are depicted by the red circles.

Light microscopy was also conducted in order to assess the impact of the thuricin CD peptides on DPC6349 cells (**Figure 5**). Compound microscope image analysis revealed that the membranes of the cells began to degrade upon exposure to the thuricin CD peptides, either individually or in combination, and this was in contrast to the negative control (PBS only) which remained relatively intact (**Figure 5**). The thuricin CD peptides resulted in the appearance of less dense regions in the membrane (red arrows) likely indicating that the membrane was being damaged due to the thuricin CD peptides. Exposure to the peptides also resulted in the emergence of smaller fragmented cells (red circles), perhaps indicative of cell lysis (**Figure 5**).

### Sequential Addition of Thuricin CD Peptides and Effects on the Membrane Potential of *B. firmus* DPC6349

**FIGURE 6:**
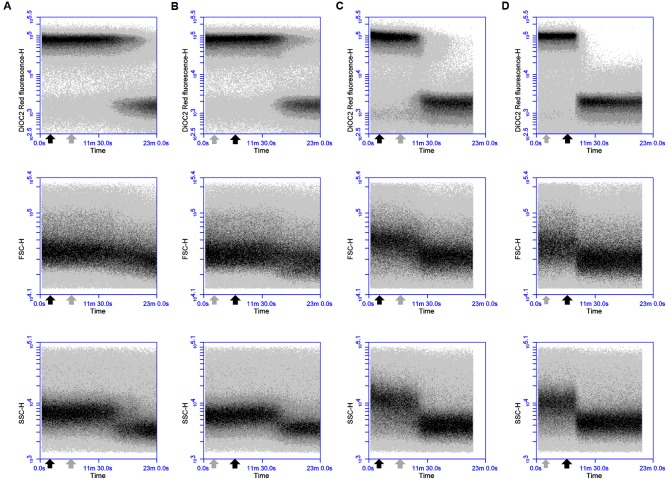
**Effects of sequential addition of equimolar concentrations of the thuricin CD peptides: The fluorochrome DiOC_2_(3) was utilized to evaluate the impact of (A)** adding 3 μM Trnα followed by 3 μM Trnβ; **(B)** 3 μM Trnβ followed by 3 μM Trnα; **(C)** 9 μM Trnα followed by 9 μM Trnβ; and **(D)** 9 μM Trnβ followed by 9 μM Trnα, on membrane potential, cell size and cell granularity. The black arrow depicts the addition of Trnα and the gray arrow represents the point of addition of Trnβ in every instance.

The antimicrobial activity of two-peptide bacteriocins is often linked to distinct roles and sequential action of both components (Wiedemann et al., 2006; Oppegård et al., 2007; Cotter et al., 2013). To test whether such a specific sequential action is also required for the activity of thuricin CD, DPC6349 cells were exposed to either Trnα (black arrow) or Trnβ (gray arrow) for 4 min before being challenged by equimolar concentrations of the second peptide (**Figures 6A–D**). At concentrations of 3 μM (1x MIC of the individual peptides), there was little or no difference between the addition of Trnα followed by addition of Trnβ, compared to the addition of Trnβ prior to Trnα (**Figures 6A,B**). Both treatments triggered depolarization and decreases in cell size and granularity approximately 13 min after the addition of the first peptide. However, when used at 3x MIC (9 μM), marginally quicker membrane depolarization and reductions in cell size and granularity were triggered by Trnβ-Trnα sequential addition (initiation of depolarization occurred approximately 6 min after the addition of the first peptide) compared to the opposite order (initiation of depolarization occurred approximately 8 min after the addition of the first peptide) (**Figures 6C,D**). These results are in stark contrast to those previously observed with lacticin 3147, where sequential addition had a major effect on activity (Morgan et al., 2005), unlike the minor differences seen here with thuricin CD peptides.

### Effects of 12 μM Thuricin CD Concentrations on the Membrane Integrity of *B. firmus* DPC6349

**FIGURE 7:**
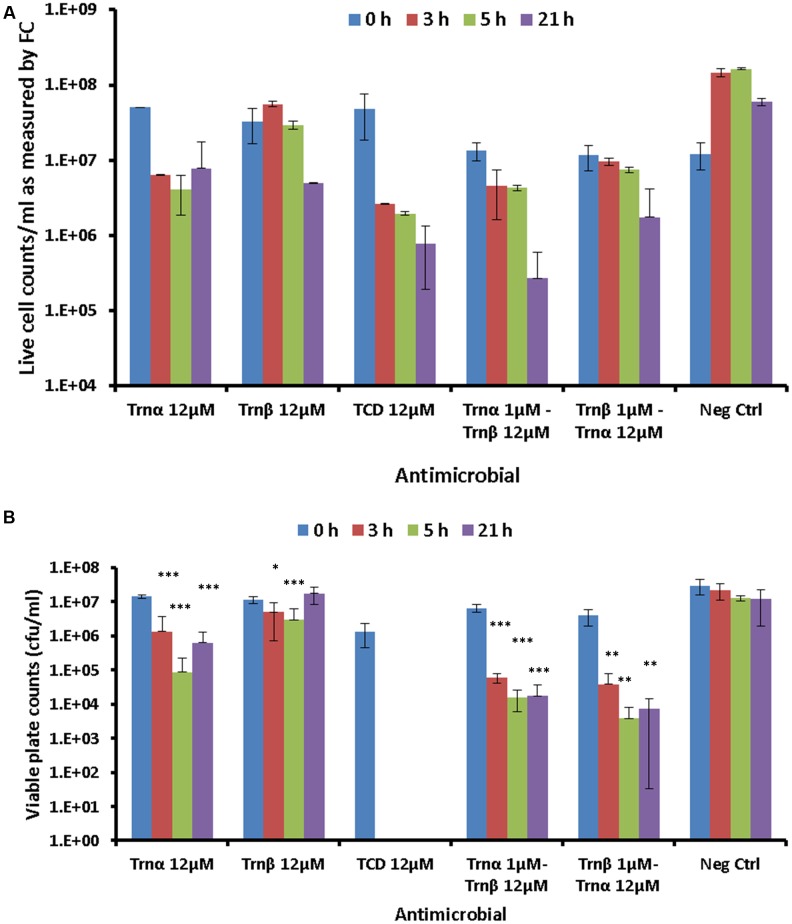
**Effects of 12 μM thuricin CD peptides on cell membrane integrity: (A)** The probes Syto 9 and PI were used to evaluate effects on the membrane integrity of DPC6349 cells by adding one of the following: 12 μM Trnα; 12 μM Trnβ; 12 μM thuricin CD; pre-incubation with 1 μM Trnα followed by the addition of 12 μM Trnβ; cells pre-incubated with 1 μM Trnβ followed by the addition of 12 μM Trnα; PBS on its own (negative control). Live cell counts as quantified by FC at time points 0, 3, 5, and 21 h are shown. **(B)** Viable counts (cfu/ml) on LB agar plates showing recovery of DPC6349 cells exposed to the conditions described above. Asterisks indicate statistically significant decreases in cfu/ml counts for each sample, relative to cfu/ml counts at T0 for each respective sample. ^∗^*P* < 0.05, ^∗∗^*P* < 0.01, ^∗∗∗^*P* < 0.001.

The effects of a lethal concentration (12 μM) of individual and combined thuricin CD peptides on the viability of DPC6349 cells were assessed by using end-point FC after staining with the membrane integrity probes Syto 9 and PI (**Figure 7A**). FC quantification of live cell counts, as determined by the number of cells excluding PI, revealed that 12 μM Trnα was more effective at causing lethal membrane damage than 12 μM Trnβ, as a greater decrease in cell counts was apparent after time points 3 and 5 h (**Figure 7A**). As expected, 12 μM thuricin CD caused greater decreases in cell counts over time, compared to equimolar concentrations of the individual peptides. The pre-incubation of cells with 1 μM Trnα prior to adding 12 μM Trnβ appeared to elicit a more pronounced killing effect, compared to cells pre-incubated with 1 μM Trnβ prior to the addition of 12 μM Trnα.

Viable plate counts in terms of cfu/ml confirmed the trends highlighted by FC analyses (**Figure 7B**). When used independently, Trnα caused significant reductions in viable cell counts after 3, 5, and 21 h (all *P* < 0.001), compared to counts at T0. In contrast, Trnβ used independently merely caused significant reductions in viable counts after 3 h (*P* < 0.05) and 5 h (*P* < 0.001) but failed to cause a decrease in viable counts after 21 h, compared to values at T0. The most drastic reduction in viable cell counts was obtained for 12 μM thuricin CD, with the failure to recover any colonies at time points 3, 5, and 21 h (all *P* < 0.05). Similarly, the pre-incubation of cells with 1 μM Trnα prior to adding 12 μM Trnβ caused significant reductions in viable counts after 3, 5, and 21 h (all *P* < 0.001). Finally, significant decreases in viable counts were also obtained for samples pre-incubated with 1 μM Trnβ prior to adding 12 μM Trnα (all *P* < 0.01). As was the case for the membrane potential findings described above, the sequential addition of the thuricin CD peptides did not appear to be an important factor governing cell viability.

## Discussion

In this study, real-time FC was utilized to monitor the time course alterations in membrane potential and physiology of sensitive *B. firmus* DPC6349 cells upon exposure to individual and combined Trnα and Trnβ peptides. The results confirm that the two peptides behave in a synergistic manner. Membrane depolarization and damage to its integrity was found to be expedited in target cells when Trnα and Trnβ acted synergistically. This finding is consistent with the 24-fold higher potency of thuricin CD, in terms of MIC value, over that of either Trnα or Trnβ alone. This synergistic activity is also shared by other two-component bacteriocins, including lacticin 3147 (Wiedemann et al., 2006). As expected, the rate of depolarization was found to be concentration-dependent, with higher concentrations of either the individual or combined peptides causing faster or greater degrees of depolarization.

We also show that the onset of membrane depolarization is concurrent with decreases in the FSC and SSC properties of the target cells. As these latter markers represent a measure of cell size (FSC) and granularity (SSC), this suggests that the thuricin CD peptides can trigger membrane depolarization, cell lysis and alterations of internal structure due to leakage of intracellular components. Such a mechanism of action would be typical of pore-forming bacteriocins such as Nisin A, which is known to cause cell lysis upon binding to the peptidoglycan precursor lipid II prior to forming pores in the membrane (Field et al., 2015). When tested, Nisin A caused depolarization and decreases in size and granularity of DPC6349 cells, as observed with the thuricin CD peptides. Since thuricin CD is a narrow spectrum bacteriocin, it is likely that a receptor other than lipid II is bound by the peptides, followed by pore formation. Such a specific receptor may be present in only a relatively small number of sensitive target species such as *C. difficile* and *B. firmus*. The precise nature of this interaction between the thuricin CD peptides and a putative unique receptor could contribute to the differences in MICs between thuricin CD and other bacteriocins such as Nisin A and lacticin 3147 against the same target strain. Furthermore, it may be the case that there are subtle differences in the extent/precise rate of leakage of ions across the membrane and consequent depolarization and cell death caused by different bacteriocins, resulting in differences in the MIC against the same target.

In contrast to the bacteriocins we investigated, the potassium ionophore, valinomycin, did not elicit any decrease in cell size and granularity despite causing very rapid membrane depolarization. Furthermore, light microscopy analyses showed the presence of shorter rods in DPC6349 cells after 1 h of exposure to thuricin CD peptides, whereas small and apparently fragmented cells, resembling remnants of lysed cells, were detected after 5 h of treatment. Altogether, these results led us to hypothesize that the mode of action of thuricin CD involves pore formation, eliciting membrane depolarization due to the influx/efflux of ions across the membrane and ultimately resulting in lysis of the target cells.

To provide further support for this hypothesis, sequential addition studies were carried out whereby DPC6349 cells were initially exposed to valinomycin before being challenged with either the individual peptides or combinations of the thuricin CD peptides. Valinomycin alters only the membrane potential component of the PMF by facilitating the movement of K^+^ ions from areas of high to low concentration (Bruno et al., 1992; Maftah et al., 1993; Lamsa et al., 2012). Under conditions of high (150 mM) extracellular K^+^ ions, sub-lethal concentrations (156 nM) of valinomycin triggered an extremely swift and large depolarization in DPC6349 cells, most likely due to a valinomycin-induced transfer of these K^+^ ions toward the negatively charged intracellular environment. Within a few minutes, DPC6349 cells counteracted this depolarization and rapidly restored their basal membrane potential, presumably by activating ATPase efflux pumps to extrude excess K^+^ ions. Addition of thuricin CD again caused cell depolarization, which this time appeared to be irreversible, even after further supplementation with valinomycin. This suggests that thuricin CD causes a rapid loss of membrane functionality and/or integrity in DPC6349 cells, which are thus unable to restore the ionic equilibrium at both sides of the membrane. This mechanism is generally associated with the antimicrobial activity of pore-forming bacteriocins (Wiedemann et al., 2006; Field et al., 2010, 2015; Noll et al., 2011). A similar ability to cause permanent membrane depolarization was also observed with Trnα and Trnβ alone, indicating that each of the individual peptides may also be able to form pores. Such permeabilization of the cell membrane and reduction in membrane potential, due to transport of ions across the membrane, has also been reported for the bacteriocin mesentericin Y105 (Maftah et al., 1993). Interestingly, a separate study involving another sactibiotic, subtilosin A, revealed that a ‘buried orientation’ adopted by the subtilosin A peptide upon interaction with the membrane triggers conformational changes in the lipid bilayers causing membrane permeabilization (Thennarasu et al., 2005). Due to the relatively hydrophobic nature of the thuricin CD peptides, it is plausible that the hydrophobic residues of Trnα and Trnβ also interact with the lipid bilayers in membranes of target cells akin to subtilosin A. An interaction of this nature between the thuricin CD peptides and the hydrophobic membrane could result in alterations of the shape of the membrane, facilitating pore formation, consequent depolarization and eventual cell death.

Kinetic FC analyses were also employed to ascertain the real-time changes in membrane potential caused by the sequential action of thuricin CD peptides in this study. By exposing the cells to either one of the peptides for a short duration of time followed by the addition of the second peptide, we ultimately aimed to evaluate whether the antimicrobial activity of thuricin CD is linked to the specific role and sequential action of its components, as reported for other two-peptide bacteriocins (Wiedemann et al., 2006; Oppegård et al., 2007; Cotter et al., 2013). At 3x MIC of the individual peptides, a marginally faster effect on depolarization and consequent decreases in cell size and granularity was observed when Trnβ was added prior to Trnα, as compared to the reverse order. However, Trnα appeared to cause a slightly sharper decrease in cell size relative to the steadier reduction elicited by Trnβ, suggesting that Trnβ could be more efficient at eliciting depolarization of the membrane whereas Trnα may be faster at triggering cell lysis. Nonetheless, the differences between the sequential addition experiments were extremely small. Viable counts obtained by both FC analysis of PI-negative cells and traditional plating methods also confirmed that 12 μM Trnα had a marginally faster killing effect than equimolar concentrations of Trnβ. The pre-incubation of cells with sub-lethal concentrations of one of the peptides followed by the addition of higher amounts of the second peptide revealed that there was little difference between the treatments in terms of the sequential addition of the peptides, when assessed with membrane integrity probes. Pre-incubation of cells with higher concentrations would likely have resulted in cell lysis and cell death, precluding the precise assessment of the effects of sequential addition of the peptides. Unlike our membrane potential experiments which permitted the sequential addition of the thuricin CD peptides in real-time, our membrane integrity sequential addition assays were reliant on the capacity of the individual peptides to adsorb to the cell surface before being washed off with PBS to remove any unbound peptide. A marked decrease in cfu/ml was found for cells which were pre-incubated with sub-lethal concentrations of one of the peptides followed by the addition of higher concentrations of the second peptide. Notably, cells treated with 12 μM thuricin CD failed to recover and form colonies on agar plates. The failure of colonies to recover even upon transfer to rich media may be due to the bactericidal effects and extensive cell lysis caused by the synergistic interactions of the thuricin CD peptides. It is plausible that Trnα and Trnβ target very similar or almost identical receptors in the membrane. It may be the case that binding of one of the thuricin CD peptides to the receptor triggers a conformational change in the receptor and/or nearby proteins or structures. A potential conformational alteration of this nature may expose the binding site of another receptor, facilitating recruitment of the other thuricin CD peptide. All these effects may lead to faster and/or a greater degree of pore formation when the two peptides potentiate each other’s effects.

In conclusion, this is the first study conducted to gain initial insights into the mechanism of action of the sactibiotic thuricin CD. The similar structure of the two thuricin CD peptides, their potent activity against Gram positive spore-formers and the observations from this study which indicate that sequential addition of the peptides has negligible effects, point to a unique mechanism of action for these peptides. Furthermore, the extremely narrow spectrum of activity and the lack of resistant mutants (Rea et al., 2010) may indicate that the receptor for thuricin CD could be an essential protein/structure that is present in *B. firmus* and *C. difficile* and unique to certain spore-forming cells and absent in non-target species. Mutation/disruption of such an essential receptor/protein in target cells likely precludes the isolation of any resistant mutants. Due to the narrow-spectrum activity of thuricin CD, it is highly unlikely that lipid II is a target receptor for thuricin CD. Thus, while we have not identified a specific receptor/binding site here or elucidated the precise nature of the synergistic interactions of the peptides, this study nonetheless reveals for the first time that both Trnα and Trnβ have the ability to cause alterations of the membrane, and that the onset of depolarization triggered by the pore-forming ability of thuricin CD peptides is accompanied with decreases in cell size and granularity, indicating cell lysis.

## Conclusion and Perspectives

Elucidating the mechanism of action of bacteriocins is a key step in potentially utilizing them for therapeutic purposes. Since thuricin CD is a narrow-spectrum bacteriocin with potent activity mainly against *C. difficile* strains, it has great potential for use as an alternative therapeutic option to treat *C. difficile* infection (CDI), which is often caused by extensive damage to the gut microbiota due to the use of broad-spectrum antibiotics. Here, we used a thuricin CD-sensitive *B. firmus* strain as an alternative host to study the effects of thuricin CD on the physiology of sensitive bacterial cells. The data presented here demonstrates that thuricin CD inflicts significant damage on the cell membrane and that depolarization of the membrane is likely to be a critical step in this process. By employing the use of FC to gain insights into thuricin CD’s impact on membrane potential and membrane integrity, in conjunction with traditional cell viability assays, we conclude that depolarization causes decreases in cell size and cell complexity and this is likely to be due to the lytic action of the thuricin CD peptides.

## Author Contributions

HM, VF, MR, CH, PC, and RR designed the study and wrote the manuscript. HM, VF, and PO conducted the experiments, interpreted and analyzed the data. All authors read and approved the final manuscript.

## Conflict of Interest Statement

The authors declare that the research was conducted in the absence of any commercial or financial relationships that could be construed as a potential conflict of interest.

## Abbreviations

BD: Becton Dickinson
BHI: brain heart infusion
CDI: *Clostridium difficile* infection
cFDA: carboxyfluorescein diacetate
cfu: colony forming units
DiBAC_4_(3): Bis-(1,3-Dibutylbarbituric acid) Trimethine Oxonol
DiOC_2_(3): 3,3′-diethyloxacarbocyanine iodide
DMSO: dimethyl sulfoxide
DPC: dairy products culture collection
FC: flow cytometry
IPA: isopropanol
LB: Luria Bertani
MIC: minimum inhibitory concentration
OD: optical density
PBS: phosphate buffered saline
PI: propidium iodide
RP-HPLC: reverse-phase high performance liquid chromatography
TFA: tri-fluoroacetic acid.
